# Characterization of polydactyly-derived chondrocyte sheets versus adult chondrocyte sheets for articular cartilage repair

**DOI:** 10.1186/s41232-017-0053-6

**Published:** 2017-11-01

**Authors:** Miki Maehara, Masato Sato, Eriko Toyoda, Takumi Takahashi, Eri Okada, Tomomi Kotoku, Masahiko Watanabe

**Affiliations:** 1 Department of Orthopaedic Surgery, Surgical Science, Tokai University School of Medicine, Kanagawa, Japan; 2 CellSeed Inc., Tokyo, Japan

**Keywords:** Cartilage regeneration, Cell sheet technology, Chondrocyte sheet, Osteoarthritis, Polydactyly-derived chondrocyte

## Abstract

**Background:**

We previously conducted a first-in-human clinical study of articular cartilage repair using autologous chondrocyte sheets and confirmed the regeneration of hyaline-like cartilage in all eight patients. However, regenerative medicine with autologous chondrocyte sheets requires the harvesting of tissue from healthy regions, and the quality of this tissue varies between individuals. To overcome such limitations, allogeneic transplantation is a promising treatment method, particularly for articular cartilage repair. In this study, we investigated the characteristics of polydactyly-derived chondrocyte sheets fabricated from the chondrocytes of young polydactyly donors.

**Methods:**

Polydactyly-derived chondrocyte (PD) sheets were fabricated from the tissue obtained from eight polydactyly donors (average age = 13.4 months). To create these PD sheets, chondrocytes at passage 2 or 3 were seeded on temperature-responsive culture inserts and cultured for 2 weeks. For comparison, adult chondrocyte sheets were fabricated from tissue obtained from 11 patients who underwent total knee arthroplasty (TKA; average age = 74 years). To create these TKA sheets, chondrocytes and synovial cells were cocultured, and the chondrocyte sheets were triple-layered according to the protocol from our previous clinical study. Cell count, cell viability, cell surface markers, cell histology, and humoral factors secreted by the sheets were characterized and compared between the PD sheets and TKA sheets.

**Results:**

Polydactyly-derived chondrocytes proliferated rapidly to establish a layered structure with sufficient extracellular matrix and formed sheets that could be easily manipulated without tearing. Similar to TKA sheets, PD sheets expressed aggrecan and fibronectin at the protein level and the surface markers CD44, CD81, and CD90, which are characteristic of mesenchymal cells. PD sheets also produced significantly higher levels of transforming growth factor beta-1 and lower levels of matrix metalloproteinase-3 than those produced by TKA sheets, suggesting that young polydactyly-derived chondrocytes have advantages as a potential cell source.

**Conclusions:**

PD sheets exhibited characteristics thought to be important to chondrocyte sheets as well as proliferative capacity that may facilitate provision of a stable supply in the future.

## Background

Articular cartilage is composed mainly of hyaline cartilage, which exhibits viscoelastic properties. Because of its low cellularity and avascular nature, its capacity to self-regenerate after injury or degeneration is limited [1]. Existing treatment methods such as subchondral drilling [2], microfracture [3, 4], and mosaicplasty [5, 6] are all symptomatic therapies that typically fill defects with inferior fibrocartilage, which lacks the mechanical properties exhibited by native hyaline cartilage. Since being first reported by Brittberg et al. [7] in 1994, autologous chondrocyte implantation (ACI) has been performed widely as an attempt to regenerate articular cartilage. However, regeneration with fibrocartilage or with a mixture of fibrocartilage and hyaline cartilage has been reported [8], and the advantage of ACI over existing methods is controversial [9]. As such, regeneration of hyaline cartilage is a challenge for regenerative medicine and is considered important in providing a long-term treatment.

In regenerative medicine, cell sheet technology [10, 11] has been applied to the regeneration of various tissues including the cornea [12], esophagus [13], myocardium [14], and periodontal tissue [15]. Culture dishes coated with temperature-responsive polymers [16, 17] allow the collection of cells as cell sheets without the use of digestive enzymes. The collection of cells with the extracellular matrix, cell-to-cell connections, and surface proteins intact makes this technology suited to regenerative medicine.

We have applied this technology to articular cartilage repair by developing chondrocyte sheets (i.e., sheets derived from chondrocytes) for the treatment of cartilage defects. We previously reported that culturing chondrocytes on temperature-responsive culture inserts and layering three sheets formed a strong three-dimensional structure [18]. We subsequently investigated the effectiveness of such layered chondrocyte sheets for the repair of full-thickness defects in rats [19], rabbits [20], and minipigs [21] and for the repair of partial-thickness defects in rabbits [22]. Having produced such evidence, we conducted a clinical study with autologous chondrocyte sheets and treated eight patients with cartilage defects accompanied by osteoarthritis. No adverse events were detected, and improvements in both clinical scores and regeneration of hyaline cartilage were confirmed in all patients (manuscript in preparation).

However, the fabrication and transplantation of autologous chondrocyte sheets require two surgeries, and the proliferative capacity of chondrocytes also varies greatly between individuals. To overcome these issues, we investigated the possibility of using allogeneic cell sources. Allogeneic transplantation of chondrocytes is known to be immunologically tolerated, and particulated juvenile cartilage implants (De Novo NT®; Zimmer, Warsaw, IN, USA) are used clinically in the USA [23]. To ensure traceability, we focused on the surgical remains obtained from polydactyly patients at Tokai University Hospital as a source of allogeneic chondrocytes.

In this study, we collected chondrocytes from polydactyly donors and fabricated polydactyly-derived chondrocyte (PD) sheets on temperature-responsive culture inserts. For comparison, adult chondrocyte sheets were fabricated from tissue obtained from patients who underwent total knee arthroplasty (TKA), hereafter called TKA sheets. To investigate the potential of using PDs as a cell source clinically, we compared the properties of PD sheets with those of TKA sheets.

## Methods

All experiments were conducted with the approval of the Tokai University Ethics Committee and with either informed consent from the patient or parental permission.

### Fabrication of PD sheets

**Fig. 1:**
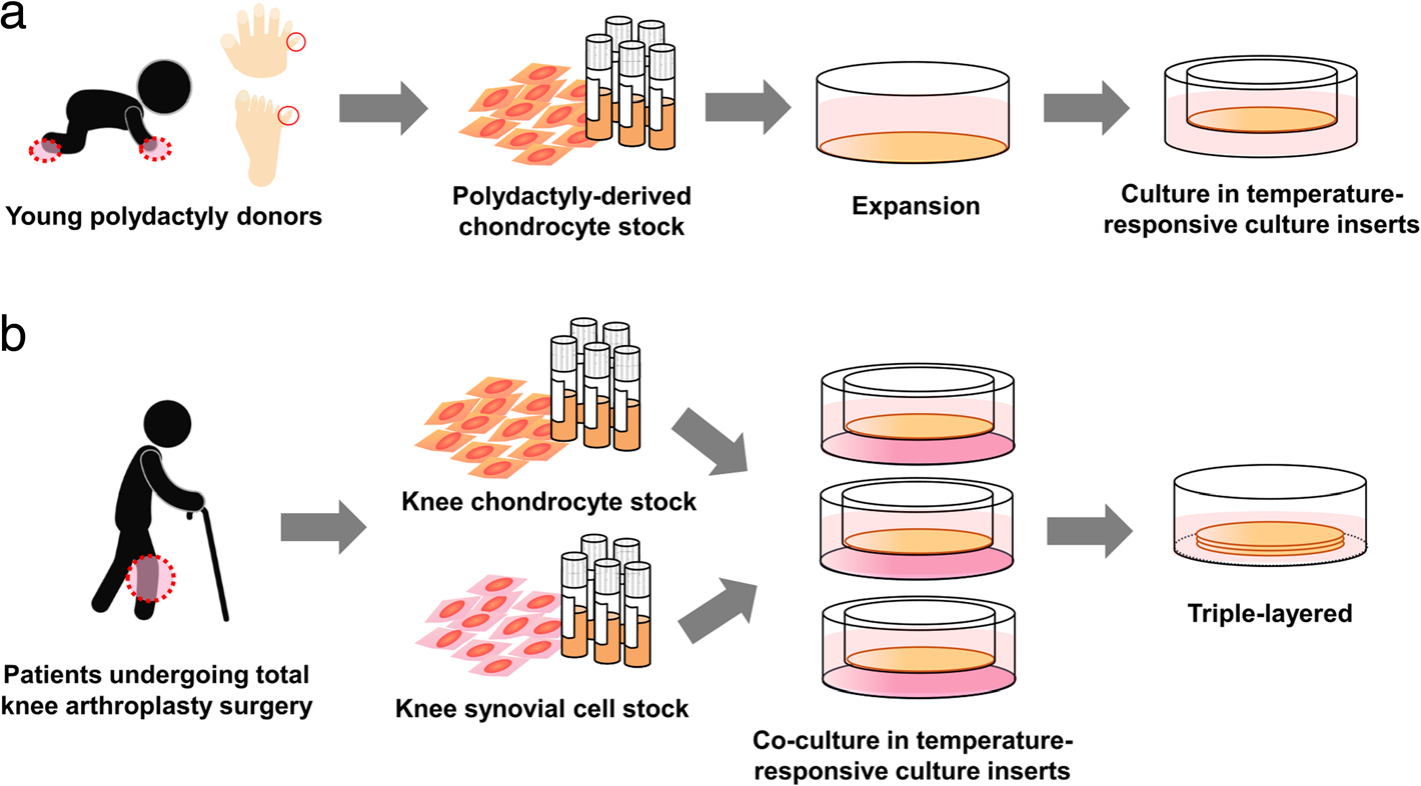
Protocol for the fabrication of PD sheets and TKA sheets. **a** Fabrication of PD sheets. PDs obtained from polydactyly surgery were passaged once or twice and stocked at − 180 °C. After further expansion, P2 or P3 cells were seeded on temperature-responsive culture inserts and cultured for 2 weeks. **b** Fabrication of TKA sheets. Adult chondrocytes and synovial cells obtained from TKA surgery were stocked at P0 and P1, respectively. Chondrocytes were seeded on temperature-responsive culture inserts and cocultured with synovial cells for 2 weeks. Three chondrocyte sheets were layered and cultured for an additional week

Cartilage tissue was obtained from eight patients (average age 13.4 months, range 8–17 months, four boys and four girls) who underwent polydactyly surgery at Tokai University Hospital. A summary of the PD sheet fabrication process is shown in Fig. 1a. Cartilage tissue was minced with scissors and subsequently incubated in Dulbecco’s modified Eagle’s medium/F12 (DMEM/F12; Gibco, Grand Island, NY, USA) supplemented with 20% fetal bovine serum (FBS; AusGeneX, Molendinar, Australia), 1% antibiotic–antimycotic solution (AB; Gibco), and 5 mg/mL collagenase type 1 (CLS1; Worthington, Mannheim, Germany) for 1.5 h at 37 °C in a humidified atmosphere of 5% CO_2_ and 95% air. The cell suspension was washed and passed through a 100-μm strainer (BD Falcon, Franklin Lakes, NJ, USA).

The collected cells were seeded at a density of 1 × 10^4^ cells/cm^2^ on six-well culture plates (Corning, Corning, NY, USA) in DMEM/F12 supplemented with 20% FBS and 1% AB and incubated at 37 °C. After 4 days, 100 μg/mL ascorbic acid (Nissin Pharmaceutical, Yamagata, Japan) was added to the medium and the medium was replaced every 3 or 4 days. Cells were passaged once or twice when they reached confluence and then cryopreserved. To fabricate PD sheets, cells were thawed and passaged once and then seeded on temperature-responsive culture inserts (CellSeed Inc., Tokyo, Japan) at 1 × 10^4^ cells/cm^2^. After 2 weeks, the culture plates were kept at 25 °C for 30 min to promote detachment of PD sheets from the inserts and the sheets were collected onto a polyvinylidene difluoride (PVDF) membrane. PD sheets were manipulated and visually confirmed to check for strength and any tearing.

### Fabrication of adult chondrocyte sheets

Adult knee articular cartilage and synovium were obtained from 11 patients (average age 74 years, range 67–79 years, five men and six women) who underwent TKA surgery at Tokai University Hospital. TKA sheets were fabricated according to previously reported methods [24, 25], which are similar to those used to create autologous chondrocyte sheets in our clinical study. A summary of the TKA sheet fabrication process is shown in Fig. 1b.

Briefly, cartilage and synovium were minced and subsequently incubated in DMEM/F12 supplemented with 20% FBS, 1% AB, and 5 mg/mL CLS1 for 4 and 2 h, respectively, at 37 °C in a humidified atmosphere of 5% CO_2_ and 95% air. The cell suspensions were washed and passed through 100-μm strainers. Chondrocytes were cryopreserved, and synovial cells were seeded at 1 × 10^4^ cells/cm^2^ and cryopreserved after confluence. To fabricate TKA sheets, chondrocytes were seeded on temperature-responsive culture inserts and cocultured with synovial cells for 2 weeks and three chondrocyte sheets were layered onto a PVDF membrane and cultured further for 1 week. TKA sheets were then manipulated and visually confirmed to check for strength and any tearing.

### Cell count and viability

PD sheets and TKA sheets were washed in Dulbecco’s phosphate-buffered saline (DPBS; Gibco). The sheets were then incubated in TripLE Express® (Gibco) at 37 °C for 15 min and centrifuged at 1500 rpm for 5 min. The cell sheets were resuspended in 0.25 mg/mL Collagenase P (Roche, Basel, Switzerland) at 37 °C for up to 30 min and then centrifuged at 1500 rpm for 5 min. The isolated cells were finally resuspended in DMEM/F12, and cell count and viability were determined using the trypan blue exclusion assay.

### Flow cytometric analysis

After obtaining the cell count, isolated cells were washed with DPBS containing 0.2% bovine serum albumin (BSA; Sigma-Aldrich, St. Louis, MO, USA) and 1 mM ethylenediaminetetraacetic acid (EDTA; Gibco). About 1.5 × 10^5^ cells were mixed in each tube with the following antibodies: hCD31–fluorescein isothiocyanate (FITC) (clone: 5.6E, Beckman & Coulter, Brea, CA, USA), hCD44–FITC (clone: G44-26), hCD45–FITC (clone: J.33, Beckman & Coulter), hCD81–allophycocyanin (APC) (clone: JS-81, BD Bioscience, Franklin, NJ, USA), and hCD90–APC (clone: 5E10, BD Bioscience). The cells were incubated for 90 min at 4 °C and then washed with DPBS containing 0.2% BSA and 1 mM EDTA. Fluoroprobe-labeled mouse IgG1 antibody (clone: 679.1Mc7, Beckman & Coulter) and mouse IgG2b antibody (clone: MG2b-57, Beckman & Coulter) were used as negative controls. Stained cells were analyzed using a FACSVerse™ cell sorter (BD Bioscience).

### Histological and immunohistochemical staining

PD sheets and TKA sheets were harvested after culture and then embedded and frozen in optimal cutting temperature compound (Sakura Finetek Japan, Tokyo, Japan). Then, sections 10 μm thick were stained for proteoglycans with Safranin O or toluidine blue using standard methods. Sections 20 μm thick were immunostained with anti-human type I collagen (COL1; 1:200; Southern Biotech, Birmingham, AL, USA), type II collagen (COL2; 1:200; Kyowa Pharma Chemical, Toyama, Japan), fibronectin (FN; 1:500; Merck, Darmstadt, Germany), and aggrecan (ACAN; 1:10; R&D Systems, Minneapolis, MN, USA) at 4 °C overnight. The sections were washed and incubated at room temperature for 1 h with the secondary antibody Alexa Fluor 488-conjugated goat anti-mouse Ig (Thermo Fisher Scientific, MA, USA) for COL2 and FN or Alexa Fluor 546-conjugated donkey anti-goat Ig (Thermo Fisher Scientific) for COL1 and ACAN. After immunostaining, the sections were washed and mounted with VECTASHIELD Antifade Mounting Medium with 4′,6-diamidino-2-phenylindole (Vector Laboratories, Burlingame, CA, USA). Microscopic images were captured under a BZ-8000 microscope (Keyence, Osaka, Japan).

### Measurement of humoral factors

A random selection of fabricated PD sheets and TKA sheets were cultured for 72 h in 3 mL of DMEM/F12 supplemented with 1% FBS and 1% AB. Supernatants were collected and centrifuged at 15,000*g* for 10 min to remove cell debris. The concentrations of transforming growth factor beta-1 (TGF-β1; R&D Systems), melanoma inhibitory activity (MIA; Roche), tissue inhibitor of metalloproteinases (TIMP1; R&D Systems), matrix metalloproteinase-3 (MMP3; Sigma-Aldrich), stanniocalcin-1 (STC1; Cusabio, College Park, MD, USA), and hyaluronan and proteoglycan link protein 1 (HAPLN1; US Biological, Salem, MA, USA) were measured using enzyme-linked immunosorbent assay (ELISA) kits. The signal detected for blank medium containing 1% FBS was subtracted to adjust for proteins contained in FBS. Measurements were repeated at least twice for each donor, and averages were used.

### Statistical analysis

Numerical results are expressed as mean and standard deviation. Statistical analysis was performed using SPSS 23.0 software (IBM Corp., Armonk, NY, USA). Differences between the two groups were identified using Student’s *t* test. The level of significance was set at *P* < 0.05.

## Results

### Isolation and proliferation of PDs

**Fig. 2:**
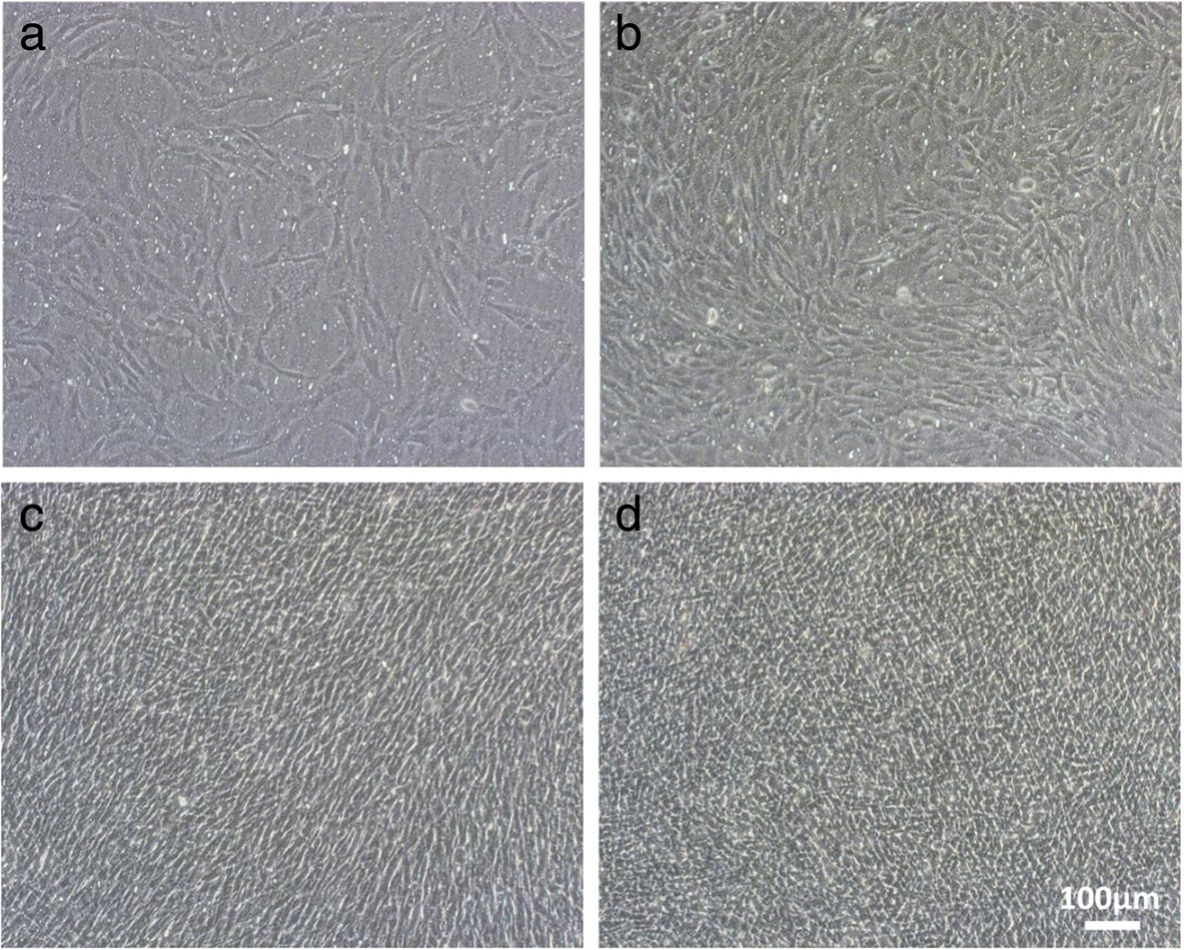
Microscopic view of the proliferation of PDs. PDs seeded on temperature-responsive culture inserts were observed on day 3 (**a**), day 5 (**b**), day 7 (**c**), and day 14 (**d**) (×100). Cells reached confluence at day 5 and, by day 14, they were tightly packed. (Scale bar = 100 μm)

The average wet weight of cartilage tissue obtained from polydactyly donors was 0.3 g, and the average number of cells collected after enzymatic digestion was 0.17 × 10^6^ cells (Table 1). Cultured cells proliferated rapidly and reached confluence around day 5 (Fig. 2b), and cells grew to an average density of 3.7 × 10^6^ cells or 22 times. Passage 1 (P1) or passage 2 (P2) cells were collected and stocked at − 180 °C. After further expansion, P1 cells proliferated 7.8 times and P2 cells proliferated 4.8 times. From the polydactyly tissue obtained from one donor, we calculated that, theoretically, 693 P2 sheets and 3326 P3 sheets can be fabricated (Table 1). The average wet weight of cartilage tissue obtained from TKA donors was 9.2 g, and the number of cells collected after enzymatic digestion was 18.5 × 10^6^ cells. However, theoretically, only 29 layered TKA sheets can be created from the P0 cells (Table 1). Furthermore, only 1 to 3 g of cartilage tissue was collected during the clinical trial; therefore, the number of autologous chondrocyte sheets that were fabricated in the clinical trial was three to seven sheets.

**Table 1:** Theoretical number of chondrocyte sheets that can be fabricated from the collected cartilage tissue

	Wet weight of harvested cartilage tissue (g)	Cell count after enzymatic digestion (× 10^6^)	Passage of cells used to fabricate sheets	Seeding cell density on temperature-responsive culture inserts	Number of layers	Theoretical number of sheets that can be fabricated
PD sheets	0.3 ± 0.2	0.17 ± 0.09	2	1 × 10^4^ cells/cm^2^	1	693
			3			3326
TKA sheets	9.2 ± 1.0	18.5 ± 6.5	0	5 × 10^4^ cells/cm^2^	3	29

*PD* polydactyly-derived chondrocyte, *TKA* total knee arthroplasty

### Cell count, sheet thickness, and macroscopic observations

**Fig. 3:**
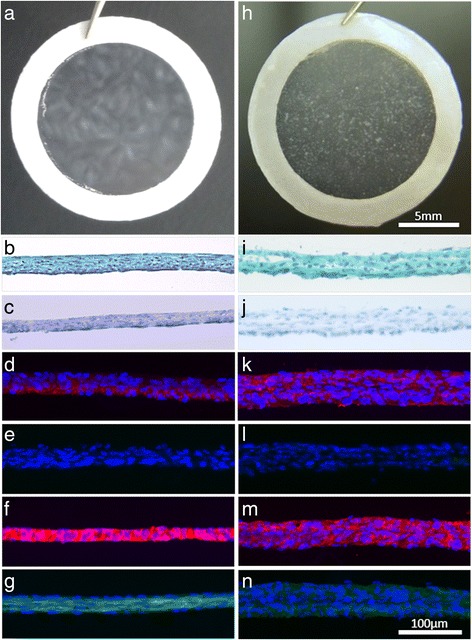
Macroscopic images and microscopic images of histological sections. Images of PD sheets (**a**–**g**) and TKA sheets (**h**–**n**). **a**, **h** Representative images of a PD sheet and TKA sheet attached to a white PVDF membrane. Histological staining for Safranin O (**b**, **i**) and toluidine blue (**c**, **j**) showed either weak or no staining for all donors (×20). Immunostaining for COL1 (red; **d**, **k**), COL2 (green; **e**, **l**), ACAN (red; **f**, **m**), and FN (green; **g**, **n**) showed positive staining for COL1, ACAN, and FN but negative staining for COL2 (×20). (Scale bar = 100 μm)

Regardless of the type of donor, all PD sheets were a high density of cells through the 2 weeks of culture and without layering (Fig. 2d). PD sheets and TKA sheets were easily harvested and manipulated without tearing (Fig. 3a, b). An average PD sheet contained 2.6 ± 0.8 × 10^6^ cells with an average thickness of 15.2 ± 4.0 μm. After a total of 3 weeks of culture, TKA sheets formed a thick structure with integrated layers (Fig. 3h). An average TKA sheet contained 1.6 ± 0.1 × 10^6^ cells with an average thickness of 45.5 ± 14.6 μm.

### Histological and immunohistochemical analyses

Histological evaluation showed that, for all donors, both PD sheets and TKA sheets stained weakly or did not stain for Safranin O or toluidine blue (Fig. 3b, c, i, j). Immunohistochemical analysis showed negative staining for COL2 and positive staining for COL1, FN, and ACAN (Fig. 3d–g, k–n).

### Flow cytometric analysis

**Fig. 4:**
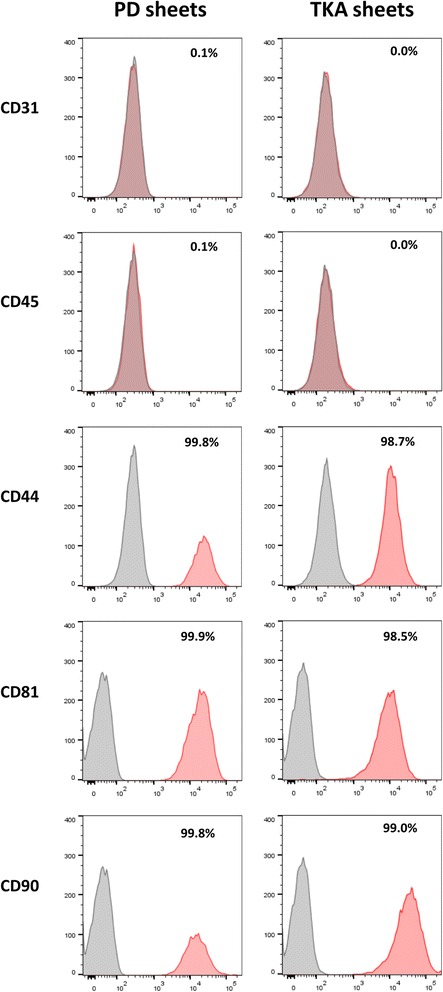
Flow cytometric analysis of surface markers. Results are shown for the flow cytometric analysis of hematopoietic cell markers CD31 and CD45 and mesenchymal stem cell markers CD44, CD81, and CD90. The target markers are indicated in red, and IgG isotype controls are indicated in gray. Both PD sheets and TKA sheets were negative for CD31 and CD45 and positive for CD44, CD81, and CD90

PD sheets and TKA sheets showed similar surface markers (Fig. 4). Both PD sheets and TKA sheets were negative for CD31 and CD45 (PD sheets: CD31 = 0.1%, CD45 = 0.1%; TKA sheets: CD31 = 0.0%, CD45 = 0.0%). Both PD sheets and TKA sheets were positive for CD44, CD81, and CD90 (PD sheets: CD44 = 99.8%, CD81 = 99.9%, CD90 = 99.8%; TKA sheets: CD44 = 98.7%, CD81 = 98.5%, CD90 = 99.0%).

### Measurement of humoral factors

**Fig. 5:**
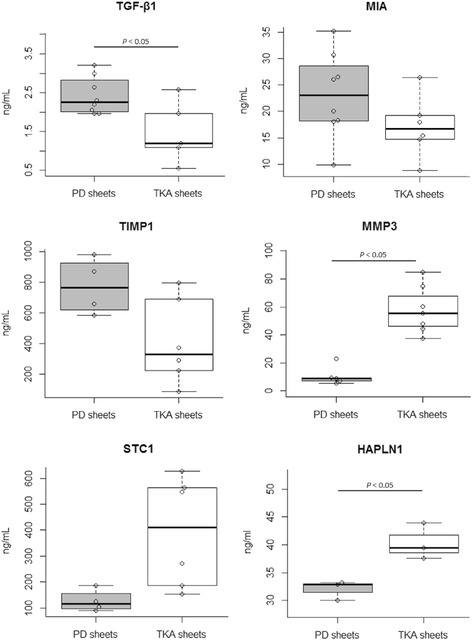
A combination of box and strip plot of the concentrations of humoral factors secreted by PD sheets and TKA sheets. Each circle indicates the average value for a single donor. Upper and lower boxes represent the 25 and 75 percentiles, lines inside the boxes are medians, upper and lower whiskers represent 1.5 times the interquartile range, and circles outside of whiskers represent outliers. PD sheets produced significantly higher concentrations of TGF-β1, and TKA sheets produced significantly higher concentrations of MMP3 and HAPLN1. *P < 0.05

The concentrations of humoral factors secreted by PD sheets and TKA sheets are summarized in Fig. 5. PD sheets produced higher concentrations of TGF-β1 (PD sheets 1.96 to 3.21 ng/mL; TKA sheets 0.55 to 2.58 ng/mL), MIA (PD sheets 9.88 to 35.15 ng/mL; TKA sheets 8.76 to 26.45 ng/mL), and TIMP1 (PD sheets 582.60 to 979.60 ng/mL; TKA sheets 83.71 to 798.10 ng/mL). TKA sheets produced higher concentrations of MMP3 (PD sheets 5.26 to 22.83 ng/mL; TKA sheets 37.29 to 84.90 ng/mL), STC1 (PD sheets 89.00 to 186.60 ng/mL; TKA sheets 153.20 to 626.60 ng/mL), and HAPLN1 (PD sheets 30.05 to 33.21 ng/mL; TKA sheets 37.59 to 43.98 ng/mL). The concentrations differed significantly between PD sheets and TKA sheets for TGF-β1, MMP3, and HAPLN1.

## Discussion

The objectives of this study were to characterize sheets fabricated from polydactyly-derived chondrocytes and to compare their properties with those of TKA sheets fabricated from adult chondrocytes. For autologous transplantation, the amount of cartilage tissue that can be collected from nonloading regions is limited, and because of the adult chondrocyte’s limited proliferative capacity, coculture with synovial cells to improve proliferation and layering of chondrocyte sheets to increase the secretion of cartilage anabolic factors [24, 26] is necessary. Our results suggest that PDs can proliferate rapidly without coculture. Theoretically, more than 600 PD sheets can be fabricated from P2 cells and more than 3000 PD sheets can be fabricated from P3 cells, whereas the number of autologous chondrocyte sheets that can be fabricated is limited. The possibility of providing a stable supply from a single lot is also attractive for ensuring the quality and safety of chondrocyte sheets.

PD sheets were found to secrete a sufficient amount of extracellular matrix and formed a layered structure during culture without having to be physically layered. This property gives an added advantage during the fabrication process of PD sheets; that is, a single layer of PD sheets, although thinner than layered TKA sheets, displayed enough strength to resist tearing and to be manipulated for transplantation. PD sheets strongly expressed ACAN and FN and were negative for CD31 and CD45, which confirmed that there was no contamination with hematopoietic cells. Similar to TKA sheets, PD sheets also expressed mesenchymal surface markers CD44, CD81, and CD90 and secreted various humoral factors related to cartilage anabolism.

Histological and immunohistochemical analyses revealed that PD sheets and TKA sheets did not stain for Safranin O, toluidine blue, or COL2. However, the autologous chondrocyte sheets that promoted hyaline cartilage repair used in our clinical study also did not stain for Safranin O, toluidine blue, or COL2 (manuscript in preparation), suggesting that chondrocyte sheets may behave differently in vivo.

The regenerative effects of chondrocyte sheets may be attributed to the protection of cartilage defects from catabolic factors within the synovial fluid, prevention of proteoglycan loss, and continued secretion of cartilage anabolic factors by transplanted chondrocytes that act as initiators of cartilage repair through the recruitment of stem cells from the bone marrow. PD sheets were found to secrete TIMP1, STC1, and HAPLN1, in addition to TGF-β1 and MIA as previously reported for TKA sheets [26]. TIMP1 inhibits the catabolic activity of MMP1 and MMP3 [27], and STC1 is involved in the regulation of angiogenesis [28] and inhibition of cartilage hypertrophy and bone formation of growth plate cartilage [29]. HAPLN1 stabilizes the association of ACAN and hyaluronan [30]. Compared with TKA sheets, PD sheets secreted significantly less MMP3, a known catabolic factor [31]. We also detected donor differences in PD sheets for humoral factor concentrations (Fig. 5), indicating that selection of donors based on the secretion levels of humoral factors may be necessary. One limitation of our study was that TKA sheets were fabricated from patients whose ages ranged from 67 to 79 years, and cartilage tissue was collected in bulk from areas that appeared normal. The autologous chondrocyte sheets used in our clinical study were fabricated from patients whose ages ranged from 30 to 59 years, and cartilage tissue was collected from the nonloading regions of knee cartilage. Although TKA sheets were shown to be effective in a rabbit xenogeneic transplantation model (manuscript in preparation), comparisons must be made carefully because TKA sheets may be inferior to those sheets used in our clinical study.

Here, we fabricated and evaluated PD sheets from P2 and P3. Current data suggest that the properties of P2 and P3 PD sheets are not significantly different (data not shown). However, the loss of cartilage properties through the passaging of PDs has been reported [32] and must be investigated further. Moreover, the in vivo characteristics of PD sheets are still under investigation. We are currently performing xenogeneic transplantation of human PD sheets into rat and rabbit osteochondral defect models to evaluate their in vivo efficacy. In addition, to make this treatment widely available off the shelf, we are investigating vitrification methods and developing systems for storing PD sheets [33, 34].

We found that PD sheets and adult TKA sheets share important characteristics, and further investigation of their in vivo efficacy will help to provide the evidence necessary to establish PDs as a cell source for allogeneic chondrocyte sheets.

## Conclusions

In this study, we characterized sheets created from PDs and compared key properties with those of TKA sheets. PDs proliferated rapidly to establish a layered structure with sufficient extracellular matrix and formed sheets that could be easily manipulated without tearing. Similar to TKA sheets, PD sheets expressed ACAN and FN at the protein level and produced significantly higher levels of TGF-β1 and lower levels of MMP3 than those produced by TKA sheets, suggesting their potential in future clinical applications.

## Abbreviations

AB: Antibiotic–antimycotic solution
ACAN: Aggrecan
BSA: Bovine serum albumin
CLS1: Collagenase type 1
COL1: Type I collagen
COL2: Type II collagen
DPBS: Dulbecco’s phosphate-buffered saline
EDTA: Ethylenediaminetetraacetic acid
ELISA: Enzyme-linked immunosorbent assay
FBS: Fetal bovine serum
FN: Fibronectin
HAPLN1: Hyaluronan and proteoglycan link protein 1
MIA: Melanoma inhibitory activity
MMP3: Matrix metalloproteinase-3
P1: Passage 1
P2: Passage 2
PD: Polydactyly-derived chondrocyte
PVDF: Polyvinylidene difluoride
STC1: Stanniocalcin-1
TGF-β1: Transforming growth factor beta-1
TIMP1: Tissue inhibitor of metalloproteinases
TKA: Total knee arthroplasty
